# Effectiveness of the national German quitline for smoking cessation: study protocol of a randomized controlled trial

**DOI:** 10.1186/s12889-022-13742-4

**Published:** 2022-07-19

**Authors:** Simone Delle, Ludwig Kraus, Simona Maspero, Oliver Pogarell, Eva Hoch, Kirsten Lochbühler

**Affiliations:** 1 IFT Institut für Therapieforschung, Munich, Germany; 2 Department of Public Health Sciences, Centre for Social Research on Alcohol and Drugs, Stockholm University, Stockholm, Sweden; 3 Institute of Psychology, ELTE Eötvös Loránd University, Budapest, Hungary; 4 Department of Psychiatry and Psychotherapy, University Hospital, Ludwig-Maximilians-Universität, Munich, Germany

**Keywords:** Smoking cessation, Telephone counselling, Randomized controlled trial, Quitline, Helpline, Tobacco smoking

## Abstract

**Background:**

Despite the decline in cigarette smoking prevalence during nearly the past two decades, tobacco use is still widespread in the German adult population, accounting for 125,000 deaths each year and causing tremendous social costs. To accelerate the reduction in tobacco smoking prevalence, evidence-based smoking cessation methods are pivotal to a national tobacco control strategy. The present study aims to evaluate the effectiveness of the national German Smokers Quitline offering cessation support to smokers.

**Methods:**

A total sample of 910 daily smokers, who are motivated to quit, will be recruited via an online access panel and randomly assigned to either the intervention (telephone counselling) or control condition. In the intervention group, participants will receive up to six proactive phone calls during an intervention period of approximately six weeks. The provided treatment will combine the principles of motivational interviewing and those of the cognitive behavioural approach to treating substance use. Participants in the control condition will receive a self-help brochure to support smoking cessation. Data collection will take place at baseline as well as three (post assessment) and twelve months (follow-up assessment) after baseline assessment. Primary outcome measures will include the seven-day point prevalence abstinence at 3-month and 12-month assessments as well as prolonged abstinence (abstinence over the 12 month period). Secondary outcome measures will include a change in smoking-related cognitions and coping strategies among all participants. Among non-abstainers, treatment success indicators such as a reduction in number of cigarettes smoked per day and changes in the number and duration of quit attempts after intervention start will be assessed. It is expected that after both three and twelve months, smoking cessation rates will be higher in the telephone counselling condition compared to the control condition.

**Discussion:**

The results will provide insights into the effectiveness of proactive telephone counselling by the national German Smokers Quitline.

**Trial registration:**

The protocol for this study is registered with the German Clinical Trials Register: DRKS00025343, Date of registration: 2021/06/07, https://www.drks.de/drks_web/setLocale_EN.do

## Background

Despite the decline in cigarette smoking prevalence over the past two decades, cigarette smoking remains the leading cause of preventable disease, disability, and death globally accounting for 8.71 million deaths in 2019 [1]. With 14.4 million current smokers in 2018, tobacco use is still widespread in the German adult population [2]. To further accelerate the reduction in tobacco smoking prevalence, federally available and effective smoking cessation interventions are needed and central to a successful tobacco control policy [3]. International efforts for tobacco interventions have been strengthened by the World Health Organization’s Framework Convention on Tobacco Control (WHO FCTC) [4]. The WHO FCTC has been developed to protect current and future generations from the health, social, and environmental consequences of tobacco use and second-hand smoke [4]. One effective measure proposed by the FCTC is the availability of telephone counselling services to support smoking cessation by trained specialists [5].

Telephone counselling services for smoking cessation are recommended because they have the potential to reach a large number of smokers [6, 7], particularly due to their flexibility in terms of time and location. They can also provide treatment to underserved groups, such as ethnic and/or linguistic minorities [8, 9], to smokers who are geographically dispersed and are attractive to smokers seeking low threshold support [10–13]. Moreover, they have the ability to tailor the intervention to key recipient characteristics and therefore offer individualized tobacco cessation to smokers [14]. Although abstinence rates achieved through telephone counselling are lower than those attained by face-to-face counselling, quitlines are effective [15] and highly cost-effective [16] due to their extraordinary reach of the smoking population [17]. For these reasons, quitlines can be considered a measure with major public health potential [18]. The effectiveness of telephone counselling for smoking cessation has been demonstrated in a systematic review [15], which shows that smokers who had called quitlines and received proactive telephone counselling were 1.38 times more likely to become abstinent than smokers who were supported by self-help materials or brief counselling at a single call (minimal intervention controls). This means that they increased their chances of long-term cessation (at least 6 months after start of the intervention) from 7 to 10%. Moreover, three to five proactive calls seem to be more effective than fewer calls [15].

Germany implemented its national Smokers Quitline in 1999. It is overseen by the Federal Centre for Health Education [‘Bundeszentrale für gesundheitliche Aufklärung, BZgA’] and offers reactive telephone counselling (calls initiated by the smoker) with a maximum of five proactive follow-up calls (calls initiated by the counsellor). Trained specialists advise smokers seeking support in smoking cessation, former smokers experiencing a relapse crisis, and information seekers who intend to quit. In 2021, more than 33,000 German smokers contacted the national Smokers Quitline. In a previous evaluation, it was found that smokers who received proactive counselling (several counselling sessions) from the national German Smokers Quitline had significantly higher abstinence rates three months after the intervention than smokers who received reactive counselling (one-time counselling) (22.3% vs. 11.1%) [19]. In this study, participants were not randomly assigned to one of the two study conditions.

As the impact of the national German Smokers Quitline could be increased by optimizing the offered intervention, the overall goal of the current study is to conduct a two-arm randomized controlled trial to evaluate the effectiveness of the national German Smokers Quitline. To accomplish this, abstinence rates will be compared among smokers, who will receive proactive telephone counselling and smokers who will receive a self-help brochure. Based on previous research [15], we expect higher smoking cessation rates among participants in the telephone counselling condition compared to the control condition (primary outcome). In addition, we hypothesize that telephone counselling will increase quit attempts, the occurrence of 24-hour abstinence, the motivation to quit, the implementation of smoking restrictions at home, and reduce daily cigarette consumption and nicotine dependence levels among non-abstainers three and twelve months after start of the intervention (secondary outcomes).

## Methods

### Study design

**Fig. 1:**
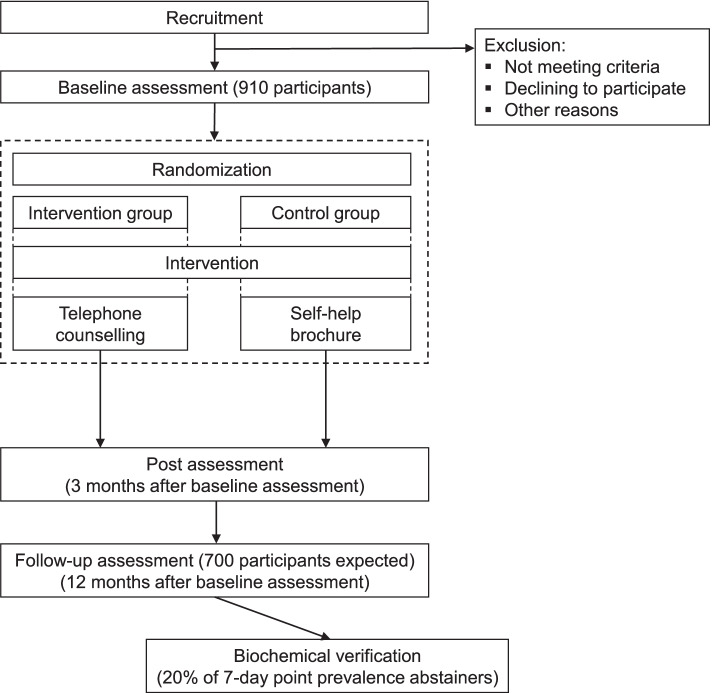
Consolidated Standards for Reporting Trials (CONSORT) diagram: study design and participant flow

The present evaluation is designed as a parallel-group, superiority, two-arm randomized controlled trial with 1:1 allocation ratio. The evaluation comprises three assessments within a period of approximately one year. Data collection will take place at baseline, three months (post assessment) and twelve months (follow-up assessment) after baseline assessment. The study design and participant flow are outlined in the Consolidated Standards for Reporting Trials (CONSORT) diagram (Fig. 1). After giving informed consent and completion of the baseline survey, participants will be randomly assigned to the intervention or control group. In the intervention group, participants will receive telephone counselling from counsellors of the national German Smokers Quitline, while in the control group they will be provided with a self-help brochure to support smoking cessation. Self-help materials were chosen as treatment in the control condition, as participants may have a benefit compared to receiving no intervention [20]. Moreover, it is considered unethical to recruit participants for a smoking cessation treatment when subsequently not offering an intervention. Providing an alternative intervention for the control group may also help achieve comparable response rates. To allow correction for any overreporting of abstinence, abstinence will be biochemically verified for a random sample of study participants who report abstinence at 12-month follow-up.

### Sample size calculation

To calculate the required sample size, a power analysis was carried out using the software G*Power [21]. Based on previous studies examining the effects of telephone smoking counselling and the use of self-help materials on seven-day-point-prevalence abstinence rates [15], a small effect size was expected. Assuming a significance level of 5% and statistical power of 0.8, the estimated required sample is *N* = 700 participants (350 participants per study condition). The calculated sample size was corrected by a 30% dropout rate [7], resulting in a total sample size of *N* = 910 enrolled participants.

### Procedure

Smokers residing in Germany will be recruited by a market research company using the PAYBACK panel, which exists since 2007. PAYBACK panelists are recruited through PAYBACK, which is the largest customer loyalty program, covering half of the German households. The panel is composed of more than 130.000 adults and is one of the largest online access panels in Germany. Panel members will be screened for eligibility by email (that contains a link to the digital questionnaire). Eligible participants will be required [1] to be at least 18 years old, [2] to have smoked cigarettes on a daily basis in the past 30 days, [3] to intend to quit smoking in the following month and [4] to provide informed consent prior to participation. Contact details of participants will be transferred to the study site twice a week. To improve adherence to the intervention protocols, referred smokers will be contacted by telephone prior to baseline assessment. During this call, they will again be informed about the procedure of the study and possible questions will be answered. Consented participants will complete the digital baseline questionnaire which will validate eligibility criteria before randomization. At least two weeks after completing the screening questionnaire, participants will either receive the self-help brochure or a call from a quitline counsellor. Three and 12 months after baseline assessment, participants will obtain another email with links to the respective questionnaires.

Participants are obliged to give study consent prior to accessing the online questionnaires. For convenience, all participants will be offered the option to complete the questionnaires via telephone interviews. Participants will receive a €25 voucher for the baseline assessment and a €15 voucher for the post and follow-up assessment respectively. For taking part in the biochemical verification, an additional remuneration of €30 will be offered. Moreover, several shopping vouchers will be raffled among participants who complete the entire study.

### Randomization

Randomization will be conducted by a research staff member. To ensure equal distribution of participants concerning selected key characteristics, randomization will be stratified based on four strata: number of cigarettes smoked per day (1–10/11–20/21–30/> 30 cigs per day), sex (female/male/diverse), age (18–30/ 31–64/> 64 years old) and level of education (low/middle/high) as reported by participants at baseline. The numbers of participants receiving each intervention are closely balanced within each stratum by performing a separate randomization procedure using permuted blocks (with each recruitment session as a block) of varying length. The first study participant per block (ordered randomly using the RAND function, Microsoft Excel) is randomized into the intervention group, the second one into the control group and so on. The randomization procedure is documented and maintained using a list which contains all possible combinations of strata and their levels as well as the number of participants in each group per strata combination. If the groups differ due to an imbalance in a certain strata combination (difference > 5 participants), the next participant with the respective strata combination will be allocated to the underrepresented group until an equal distribution is achieved.

Allocation concealment will be ensured, as participants will not be assigned to one of the study conditions until baseline measurements have been completed. Due to the nature of the intervention, neither participants nor staff can be blinded to allocation. In the study material, participants will be informed about the two study conditions. However, participants will be blinded to the study hypothesis in terms of which intervention is considered active.

### Interventions

#### Telephone counselling condition

The national German Smokers Quitline offers telephone counselling to smokers seeking support in smoking cessation, to former smokers experiencing a relapse crisis, and to information seekers. The majority of counsellors are non-academic health professionals such as nurses. Counsellors receive an intensive three-day training following the WHO training protocol “Quitline Counselling” which includes amongst other things the training on the used counselling software, the simulation of calls as well as on-site observation of experienced counsellors.

The counselling follows a structured, yet flexible counselling protocol based on the protocol of the California Smokers’ Helpline [14]. The principles of this protocol are built upon the theory of social learning [22, 23], which emphasizes the client’s capacity for self-regulation and the relevance of self-efficacy in behaviour change [14]. Therefore, the protocol combines the principles of motivational interviewing (MI) (Miller & Rollnick, 1991) and those of the cognitive behavioural approach to treating substance use e.g. [14, 24]. MI is used to induce behaviour change by enhancing the client’s intrinsic motivation to change. An empathetic, non-confrontational conversation style generates a collaborative counsellor-client relationship and allows to address the clients` ambivalence [25]. Once a client is ready to quit smoking, the counsellor applies cognitive-behavioural strategies. The client is encouraged to identify and restructure dysfunctional cognitions about smoking and quitting and to develop and implement coping strategies [14, 26]. A central tenet of this complementary approach is the counsellor’s continuous monitoring and fostering of the client’s motivation to quit and the support to increase self-management skills and self-efficacy to change [14]. Adherence to the counselling protocol is assessed on a regular basis by analyzing recorded calls and digital counselling notes taken by the counsellors.

In the telephone condition, based on their needs, participants can receive up to six proactive phone calls (14, for detailed information see 19). The first session (intake session) will take 20 to 25 minutes and will focus on assessing the client’s smoking and quitting history, their smoking habits and their motivation to quit. Moreover, the counsellor aims to resolve ambivalent feelings, to strengthen the client’s self-efficacy and motivation to quit, to identify difficult situations and to develop strategies for coping with the urge to smoke. Finally, the counsellor will encourage the client to set a quit date within the next 14 days. Participants are then offered up to five follow-up calls to support relapse prevention and the maintenance of smoking cessation. The first follow-up session will take place approximately two to three days after the quit date, the second will take place approximately seven days after the quit date; the third approximately twelve days, the fourth approximately three weeks and the fifth follow-up call approximately one month after the quit date. These sessions take about ten minutes each and cover the following topics: Assessing the client’s progress and evaluating the effectiveness of applied coping strategies, discussing withdrawal symptoms, the urge to smoke and self-efficacy as well as examining difficult situations and their handling.

#### Self-help condition

In the control condition, participants will receive the self-help brochure ‘Ja, ich werde rauchfrei!’ [‘Yes, I’ll be smoke-free’] which is being disseminated by the Federal Centre for Health Education [27]. The 92-page booklet is directed at individuals attempting to achieve smoking cessation. It follows the principles of cognitive-behavioural therapy [26] and guides the reader step by step from preparing to quit to long-term abstinence. The brochure is divided into the parts: information about smoking and smoking cessation, preparing and planning for smoking cessation and information on topics related to maintaining abstinence (e.g., anticipation of potential high-risk situations, smoking urges, developing the identity of a non-smoker, use of cognitive and behavioral coping responses, keeping weight gain in perspective, finding other forms of positive reinforcement, management of stress). Participants in the control condition will be offered telephone counselling at the end of the study.

### Outcomes

Outcome measures and covariates with their respective assessment time points are shown in Table 1.

**Table 1:** Standard Protocol Items: Recommendations for Interventional Trials (SPIRIT) diagram

	STUDY PERIOD						
	Recruitment	Enrolment	Allocation	Post-allocation			
TIMEPOINT	-t_2_	-t_1_	0	t_1_	t_2_	t_3_	t_4_
	Screening	Baseline assessment	Randomization	Intervention	Post assessment	Follow-up assessment	Biochemical verification
**ENROLMENT:**							
**Eligibility screen**	X						
**Informed consent**	X	X			X	X	X
**Randomization**			X				
**INTERVENTIONS:**							
**Telephone counselling**				X			
**Self-help brochure**				X			
**ASSESSMENTS:**							
**Socio-demographic characteristics**	X	X					
**Smoking-related variables**		X			X	X	
**Health status**		X			X		
**Received social support**					X		
**7-day point prevalence abstinence**					X	X	
**Prolonged abstinence**						X	
**Number of cigarettes smoked per day**	X	X			X	X	
**Nicotine dependence**		X			X	X	
**Number and duration of quit attempts**					X	X	
**Occurrence of abstinence of at least 24 hours**					X	X	
**Motivation to quit**	X	X			X	X	
**Implementation of smoking restrictions at home**		X			X	X	
**Smoking-related cognitions**		X			X		
**Coping strategies**		X			X		
**Use and acceptability of the received intervention**					X		
**Breath carbon monoxide (CO)**							X

Note: -t2: recruitment of participants by a market research company using a screening questionnaire; −t1: baseline assessment; 0: randomization of participants to either the intervention or control condition; t1: intervention (telephone counselling or self-help broschure); t2: post assessment 3 months after baseline assessment; t3: follow-up assessment 12 months after baseline assessment; t4: biochemical verification: 20% random sample of participants who reported 7-day point prevalence abstinence at 12-month assessment

#### Primary outcomes

In accordance with the Russell standard criteria [28], primary outcome measures will include (1) seven-day point prevalence abstinence at 3-month post and 12-month follow-up assessment, (2) prolonged abstinence (abstinence over the 12 month period allowing up to five cigarettes in total), and (3) biochemical verification of abstinence in 20% of the abstainers at 12-month follow-up. To define abstinence, cut-off scores of 8 ppm for CO will be used [29].

#### Secondary outcomes

Among participants who report being non-abstinent at 3- and 12-month follow-up, secondary outcome measures will include: (1) changes in number of cigarettes smoked per day and in nicotine dependence levels [30], (2) the number and duration of quit attempts after intervention start [31, 32], (3) the occurrence of abstinence of at least 24 hours at some point during the study (4), [31] an increase in motivation to quit (5), [33] the implementation of smoking restrictions at home [34]. A change in smoking-related cognitions (smoking outcome expectancies [35, 36]; self-efficacy to refrain from smoking [36–38]; self-efficacy for abstinence [39]) and coping strategies (avoidance of external cues [40]; perceived control over withdrawal symptoms [41]) will be assessed among all participants.

#### Covariates

The following covariates will be assessed: (1) socio-demographic characteristics (gender, age, education, employment status, nationality, marital status), (2) smoking-related variables (use of other tobacco products, years smoking, number and frequency of previous quit attempts [42], confidence in quitting and importance to quit, craving [43], other smoking household members, smoking behaviour of the partner, use of and adherence to additional supportive materials (e.g., NRT, pharmacotherapy, e-cigarettes, heat-not-burn products)), (3) health status (depressive symptoms [44], selected smoking-related illnesses, mental comorbidity), (4) received social support (supportive network and overall quitting support [45]), and (5) the use and acceptability of the received intervention (telephone counselling or self-help brochure) [31].

### Data collection and management

Participants will be recruited consecutively between October 2021 and June 2022. Therefore, data collection will take place at different time points (at baseline, three months and twelve months after baseline assessment) over a period of approximately two years. Data will be collected via online questionnaires using the software SoSci Survey [46]. It is expected that the majority of questionnaires will be administered digitally (compared to phone interviews). In order to biochemically verify abstinence, 20% of study participants who will report abstinence at 12-month follow-up and who consented to participate will be randomly selected. Research staff will meet participants at their preferred location and will collect breath carbon monoxide (CO) using a portable CO monitor.

Once a participant is enrolled, the study site will make every reasonable effort to follow the participant for the entire study period. Various procedures will be used to reduce attrition, including reminder emails and calls, flexible scheduling of the counselling sessions, and incentives for completing the questionnaires. It is expected that the rate of loss-to-follow-up will not exceed the calculated 30%. To enhance validity of data, multiple methods will be used to assess intervention adherence including the phone call prior to enrolment in order to answer possible questions and questionnaire items on the use and acceptability of the received intervention at 3-month post assessment.

All participant data is handled in accordance with the General Data Protection Regulation (GDPR). All data will be maintained confidentially before, during, and after the trial and is stored securely at the study site with access only by dedicated study team members. All substantial procedures are described in the application for the Ethics Committee and can be provided on request. The final data set will be stored for 10 years after completion of the study. A data monitoring committee is not considered necessary as participants are not blinded to the study conditions and as this is a short-term, non-invasive intervention with the opportunity to quit at any time without any negative consequences or side-effects.

### Statistical analyses

Descriptive analyses will be conducted to examine whether randomization has resulted in an equal baseline distribution of participants regarding relevant characteristics across the two conditions using *χ*^2^-tests and *t*-tests. To account for any possible group differences at baseline, confounding variables will be included in subsequent analyses. We will use logistic regression models and analyses of variance to evaluate the effectiveness of the intervention by comparing smoking cessation rates across groups. Effect sizes, as well as confidence intervals, will be reported. To analyze abstinence rates, the intention-to-treat (ITT) approach will be used. This means that data of all randomized participants will be included in the analyses unless they are deceased or have moved to an untraceable address, as recommended by the Russell Standard criteria (RS) [28]. Participants with undetermined smoking status at follow-up will be counted as smokers. In addition to the ITT principles, a complete-case analysis will be performed, in which only participants with outcome data on all assessments will be included. As all assessments use a forced entry format, no imputation for missing values will be needed. All analysis will be conducted using the statistical program R and R Studio [47, 48].

## Discussion

This study protocol presents the design of a two-arm randomized controlled trial with baseline, post and follow-up assessments to examine the effectiveness of proactive telephone counselling for smoking cessation by the national German Smokers Quitline. A major aim of the national German Smokers Quitline is to provide free counselling to smokers who want to quit smoking and thereby decrease smoking rates in the German population.

In the intervention condition, smokers will receive counselling from the national German Smokers Quitline, while in the control condition, they will receive a self-help brochure to support smoking cessation. Both interventions are based on the principles of cognitive-behavioural therapy [26], however, smokers in the intervention condition can receive structured but tailored counselling in multiple sessions up to six weeks length. Based on previous research [15, 31], it is expected that smoking cessation rates will be higher in the intervention than in the control condition three as well as twelve months after the intervention. In addition, we hypothesize that telephone counselling will increase quit attempts, the occurrence of 24-hour abstinence, the motivation to quit, the implementation of smoking restrictions at home, and reduce daily cigarette consumption and nicotine dependence levels among non-abstainers.

The current study is the first to examine the effectiveness of the national German Smokers Quitline using a randomized design with post and follow-up assessments allowing to test the short- and long-term effects of the intervention. The assessment of abstinence at twelve-month follow-up is recommended as it is more closely related to life-long abstinence and false-positive results are less likely [49]. Another strength of the current study lies in the application of RS criteria for trials of cessation aids [28, 50], which enhances the quality of the data and the generalizability of the results. One limitation of this study is the assessment of abstincence via self-reports, although self-reports have previously been shown to be reliable and valid measures of respondents’ smoking behaviour [51]. Additionally, in a subsample of all study participants who report 7-day point prevalence at follow-up, smoking cessation will be biochemically verified to counteract reporting biases [29, 31]. Another limitation of this study is that participants will be recruited via an online panel, which may limit the generalizability of study results.

Quitlines are considered an important and cost-effective population-based tobacco control strategy [17]. However, of the 19.9% of German current and recent former smokers who try to quit smoking per year, only 13.0% use at least one evidence-based method to support their cessation attempt. The majority of smokers does not make use of quitlines; they reach only about 0.8% of individuals who make a quit attempt [52]. If the intervention provided by the national German Smokers Quitline is found to be effective, awareness should be enhanced to exploit the proportion of smokers that are advised to stop smoking through telephone counselling. Its population-based impact, which means an increased use of the quitline services, could be expanded by communication campaigns. The recruitment for quitline services can be further enhanced by targeting populations that have a high tobacco use prevalence [17]. The results of the current study will provide insights into the effectiveness of the national German Smokers Quitline.

## Data Availability

Anonymized study data and statistical codes to analyses may be made available on request from the corresponding author following study closure.

## Abbreviations

BZgA: Bundeszentrale für gesundheitliche Aufklärung, Federal Centre for Health Education
CO: Carbon monoxide
CONSORT: Consolidated Standards for Reporting Trials
DGPs: Deutsche Gesellschaft für Psychologie, German Psychological Society
DRKS: Deutsches Register klinischer Studien
FCTC: Framework Convention on Tobacco Control
GDPR: General Data Protection Regulation
IFT: Institut für Therapieforschung
MI: Motivational interviewing
NRT: Nicotine replacement therapy
RS: Russell Standard
SPIRIT: Standard Protocol Items: Recommendations for Interventional Trials
WHO: World Health Organization
